# The Association between Endometriosis, Tubal Ligation, Hysterectomy and Epithelial Ovarian Cancer: Meta-Analyses

**DOI:** 10.3390/ijerph13111138

**Published:** 2016-11-14

**Authors:** Chunpeng Wang, Zhenzhen Liang, Xin Liu, Qian Zhang, Shuang Li

**Affiliations:** 1 School of Mathematics and Statistics, Northeast Normal University, Changchun 130024, China; 2 Epidemiology and Statistics, School of Public Health, Jilin University, Changchun 130021, China; liangzz15@mails.jlu.edu.cn (Z.L.); xliu@jlu.edu.cn (X.L.); Zhangqian15@mails.jlu.edu.cn (Q.Z.); lishuang900603@163.com (S.L.)

**Keywords:** epithelial ovarian cancer, endometriosis, hysterectomy, tubal ligation, meta-analysis

## Abstract

To investigate the association between endometriosis, tubal ligation, hysterectomy and epithelial ovarian cancer. Relevant published literatures were searched in PubMed, ProQuest, Web of Science and Medline databases during 1995–2016. Heterogeneity was evaluated by I^2^ statistic. Publication bias was tested by funnel plot and Egger’s test. Odds ratio and 95% CI were used to assess the association strength. The statistical analyses in this study were accomplished by STATA software package. A total of 40,609 cases of epithelial ovarian cancer and 368,452 controls in 38 publications were included. The result suggested that endometriosis was associated with an increased risk of epithelial ovarian cancer (OR = 1.42, 95% CI = 1.28–1.57), tubal ligation was associated with a decreased risk of epithelial ovarian cancer (OR = 0.70, 95% CI = 0.60–0.81), while hysterectomy show no relationship with epithelial ovarian cancer (OR = 0.97, 95% CI = 0.81–1.14). A stratified analysis showed there were associations between endometriosis and the increased risk of epithelial ovarian cancer for studies conducted in USA and Europe. Meanwhile, there were associations between tubal ligation and the decreased risk of epithelial ovarian cancer for studies conducted in USA, Asia, Europe and Australia. The result indicated that endometriosis was a risk factor of epithelial ovarian cancer whereas tubal ligation was a protective risk factor of epithelial ovarian cancer, hysterectomy may have no relationship with epithelial ovarian cancer.

## 1. Introduction

Epithelial ovarian cancer (EOC) is the fifth leading cause of cancer death and the eighth most common cancer in women [1,2]. Approximately 1 in 70 women in the United States bear the pain of EOC, and the annual fatality rate of EOC cases is about 50% [3]. Clinical and epidemiologic studies have shown that the early detection technology of EOC is not yet perfect, and EOC is often diagnosed at advanced stage. In addition, overall five-year survival rate of EOC is about 45% [4]. Thus, the exploration of risk factors is imperative for the reduction of the burden of disease.

Recently, certain modifiable risk factors have been reported to be related to EOC. Ness et al. demonstrated that endometriosis was a risk factor of EOC [5]. However, in some studies, endometriosis may be not a risk factor of EOC [6,7]. Several years ago, most studies have proved that hysterectomy with conservation of at least one ovary could decrease the risk of developing EOC by 20%–40% [8]. However, recent studies have reported that hysterectomy was a risk factor of EOC. Tubal ligation is also associated with a 30% decreased risk of EOC incidence [9,10].

Although many researches about the associations of endometriosis, hysterectomy, tubal ligation and EOC based on individual-patients or pooled analyses based on published studies have already been conducted, such as Pearce et al. 2012 on endometriosis, Jordan et al. 2013 on hysterectomy, and Cibula et al. 2011 on tubal ligation [8,10,11], several years past, new studies were published, so, in our study, we re-researched the individual-patients studies and included several new studies after 2013 which added 14,424 cases and 265,720 controls [6,7,12,13,14], and then conducted an updated meta-analysis which further explore and confirm whether endometriosis, hysterectomy, tubal ligation are associated with EOC. In our study, 38 case-control publications from 1988 to 2016 have been brought together, checked, and analyzed centrally.

## 2. Methods

### 2.1. Literature Search

The medical literature electronic databases of PubMed, ProQuest, Web of Science (SCI) and Medline were searched for case-control studies which were related to risk factors of EOC and published prior to September 2016. The terms (endometriosis and epithelial ovarian cancer); (hysterectomy and epithelial ovarian cancer); (tubal ligation and epithelial ovarian cancer) and (endometriosis or hysterectomy or tubal ligation and epithelial ovarian cancer) of search strategy were used. In addition, references of each included studies were manually searched to identify any additional studies that were not indexed by the electronic database.

### 2.2. Study Selection

The following inclusion criteria were used in selecting case-control studies: (1) studies in which a case-control study design were used and investigated the association between endometriosis or hysterectomy or tubal ligation and EOC; (2) cases consisted of women who were histological diagnosed EOC; (3) the data of case and control numbers in different risk factors were presented in the publication or the data given were necessary to calculate these; (4) Odds ratio (OR) or OR with 95% confidence intervals (CIs) of each risk factor were presented in the publication or the data given were necessary to calculate these; (5) when the publications were from the same or overlapping data, we selected the most recent or largest population; (6) English was stipulated as the publication language; (7) reviews, letters or case reports were excluded.

### 2.3. Data Extraction

According to the predefined selection criteria, the data in whole process was independently extracted from each study by two reviewers. When disagreements appeared, the reviewers reanalyzed the study and resolved disagreements by discussion. For each study, we recorded number of case and control, the first author’s name, year of diagnosis, year of publication, study geographic region, confounders and study type as the basic contents of the data extraction. If the number of case and control in different risk factors were available, we abstracted directly, or we calculated via raw data given in the articles.

### 2.4. Quality Assessment

We evaluated the quality of individual studies using the Newcastle-Ottawa Scale (NOS) [15]. The NOS consists of three broad perspectives of quality: the selection, compare ability and exposure of each case-control study. The NOS assigned a maximum of four points for selection, two points for comparability and three points for exposure [16]. In this study, we considered a study with a total of seven points or greater as a high-quality study. In this meta-analysis, of all case-control studies involved, 36 (95%) were of high quality with an average NOS score of 7.4. Any disagreements were resolved by discussion and re-evaluation of the methodology of the study with a third reviewer.

### 2.5. Statistical Analysis

All statistical analyses in this study were performed with STATA software package (version 12.0, StataCorp., College Station, TX, USA). OR and OR with 95% CI were used as measures of the association strength. We evaluated the degree of heterogeneity in eligible studies by using I^2^ statistic. If *p* < 0.10, statistically significant heterogeneity was indicated, then random-effect models were conducted for the heterogeneous data. Otherwise, fixed effects model were used. Publication bias was evaluated via funnel plot and Egger’s test, with *p* < 0.05 indicating statistical significance. Subgroup analyses were conducted based on area. A cumulative meta-analysis was conducted to reflect a framework for the dynamic change trend for the results of the study and measure the effect of each research as evidence accumulates and find the trend in estimated risk effect [17]. In this meta-analysis, studies were estimated by year of publication.

## 3. Results

### 3.1. Search Results and Study Characteristics

**Figure 1:**
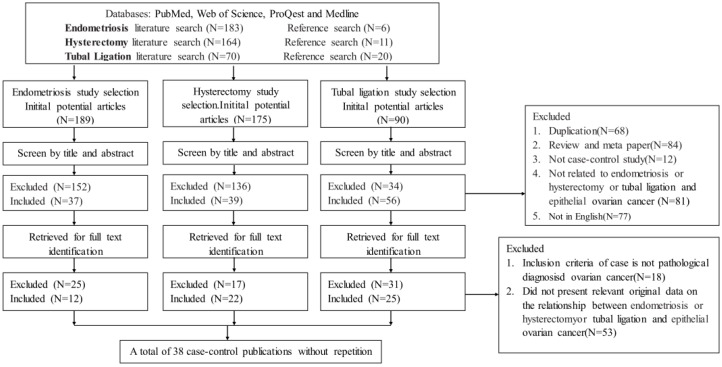
The process of study selection.

As shown in Figure 1, we identified 12 studies [5,6,7,11,14,18,19,20,21,22,23,24] for the examination of endometriosis and epithelial ovarian cancer, 22 studies [11,12,14,18,19,22,24,25,26,27,28,29,30,31,32,33,34,35,36,37,38,39] for the examination of hysterectomy and epithelial ovarian cancer, 25 studies [6,7,9,11,13,14,18,19,21,24,30,31,33,34,37,38,39,40,41,42,43,44,45,46,47] for the examination of tubal ligation and epithelial ovarian cancer. A total of 38 eligible case-control publications during the period 1988 and 2016 were identified in this meta-analysis. The main characteristics of the eligible 38 publications were listed in Table 1. All included studies were composed of 40,609 cases of ovarian cancer and 368,452 controls, the age range of all subjects was from 20 to 74 years old. The 38 publications were done in 11 countries altogether, mainly in North America, Europe, Oceania and Asia. Although there were several large prospective (cohort) studies that have published to show the association between endometriosis, tubal ligation, hysterectomy and epithelial ovarian cancer [13,48,49,50], for consistency we chose to restrict our analysis to results from case-control studies.

### 3.2. Quantitative Synthesis

All analyses of each risk factor were based on the random-effects model (for endometriosis, *p* of heterogeneity was ˂0.001; for hysterectomy, *p* of heterogeneity was ˂0.001; for tubal ligation, *p* of heterogeneity was ˂0.001). High heterogeneity were found among the included studies of each risk factor. Considered that the 38 publications we included were mainly done in North America, Europe, Oceania and Asia, and we wanted to compare the different association among these areas, so we conducted a subgroup analysis based on area. Table 2 displayed the number of subjects and ORs with 95% CI for endometriosis, hysterectomy, tubal ligation and ovarian cancer in different areas.

A statistical-significant association was found between endometriosis and EOC (OR = 1.42, 95% CI = 1.28–1.57). For area, there were associations between endometriosis and the increased risk of ovarian cancer for North America (OR = 1.42, 95% CI = 1.27–1.60), Europe (OR = 1.28, 95% CI = 1.12–1.47) and Oceania (OR = 2.52, 95% CI = 1.63–3.90) (forest plots of subgroup analysis are shown in supplement Figure S1).

No statistical association was found between hysterectomy and EOC risk (OR = 0.97, 95% CI = 0.81–1.14). For area, there were no associations between hysterectomy and EOC for North America (OR = 1.00, 95% CI = 0.93–1.09), Europe (OR = 0.97, 95% CI = 0.86–1.10) and Oceania (OR = 0.98, 95% CI = 0.79–1.21).

There was a significant association between tubal ligation and a decreased risk of EOC (OR = 0.70, 95% CI = 0.60–0.781). For area, tubal ligation was associated with a decreased risk of EOC for North America (OR = 0.68, 95% CI = 0.64–0.72), Oceania (OR = 0.48, 95% CI = 0.39–0.60) and Europe (OR = 0.82, 95% CI = 0.75–0.89). But there was no association between tubal ligation and EOC for Asia (OR = 0.85, 95% CI = 0.58–1.24).

### 3.3. Bias Diagnosis

**Figure 2:**
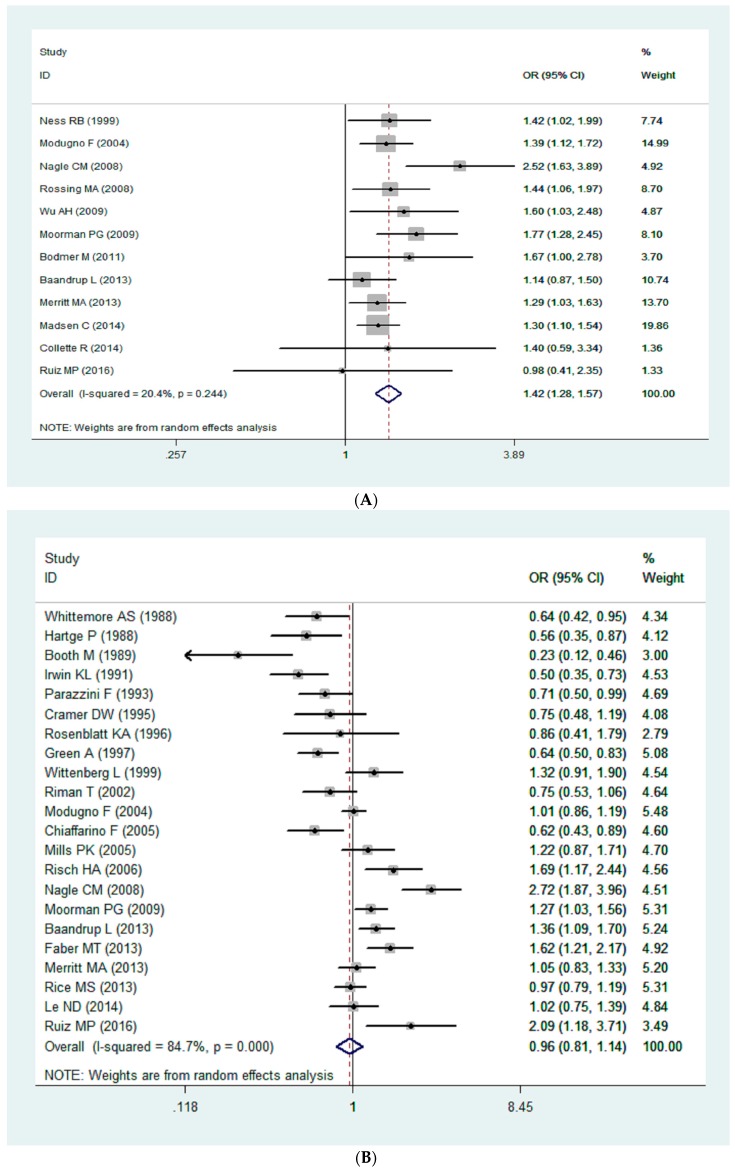

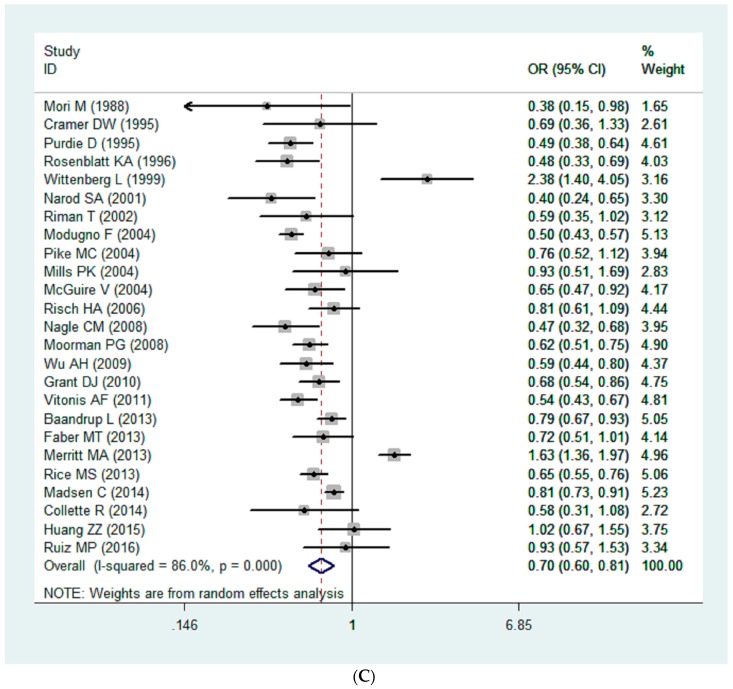
(**A**) The forest plot for the association between endometriosis and EOC; (**B**) The forest plot for the association between hysterectomy and EOC; (**C**) The forest plot for the association between tubal ligation and EOC.

Funnel plots and Begg’s test were used to assess publication bias. The result of funnel plots for each risk factor suggested no obvious asymmetry (The figures were shown in supplement Figure S2). Then the Egger’s test was used to confirm the results (for endometriosis: *p* = 0.242; for hysterectomy: *p* = 0.314; for tubal ligation: *p* = 0.757). Neither funnel plots nor Egger’s test suggested any obvious evidence of publication bias (Figure 2).

### 3.4. Sensitivity Analysis

In the sensitivity analysis, when all studies was removed one by one and the rest were analyzed sequentially by meta-analysis, the pooled ORs were not materially altered with the overall pooled ORs, indicating our results were statistically robust (shown in Supplementary Materials Figure S3).

### 3.5. Cumulative Meta-Analysis

In cumulative meta-analysis, the estimates gradually became consistent, a more significant association between the three factors and risk of EOC was observed as evidence accumulated by publication year (shown in Supplementary Materials Figure S4).

## 4. Discussion

According to the inclusive papers, which included 40,609 cases of epithelial ovarian cancer and 368,452 controls, we found that endometriosis was a risk factor of epithelial ovarian cancer whereas tubal ligation was a protective risk factor of epithelial ovarian cancer, hysterectomy may had no relationship with epithelial ovarian cancer.

The previous meta-analysis have reported that endometriosis is conformed to be a risk factor of EOC. Pearce CL et al. reported that endometriosis was associated with a significantly increased risk of clear cell (OR = 3.05, 95% CI = 2.43–3.84) and endometrioid invasive ovarian cancers (OR = 2.04, 95% CI = 1.67–2.48) [11]. The combined outcomes in this meta-analysis suggested that endometriosis was a risk factor of EOC (OR = 1.42, 95% CI = 1.28–1.57), which was consistent with the above-mentioned reports. In the study of Pearce et al., the data was also extracted from case-control studies, but the standard of subgroup analyses and confounding factor were different from our study. Although the data sources and the object of the above study was different from our study, we got the same conclusion, confirming that endometriosis is a quite possibly risk factor of EOC. Endometriosis is a common gynecological diseases, which can leads to dysmenorrhea, pelvic inflammation, adhesions and infertility [51]. The pathological features of endometriosis are ectopic growth of endometrial glands and stroma and the growth of endometrial tissue is oestrogen dependent [52]. Recent researches reviewed the mechanisms of endometriosis in EOC mainly in three ways. One was an oestrogen-dependent way. Ness et al. reported that endometriosis was a precursor for EOC and was more easier developed to EOC in the condition of low- progesterone and high-oestrogen [51]. The second way was genetic mutations in endometriosis tissues, such as hepatocyte nuclear factor-1β (HNF-1β) [53] and ARID1A [12]. Moreover, chronic inflammation, heme or free iron-induced oxidative stress in endometriosis tissues also reported related to EOC [13].

As for the other risk factor, hysterectomy, Jordan et al. found that hysterectomy decreased the risk of EOC (OR = 0.81; 95% CI = 0.72–0.92) [8]. However, the outcomes in this meta-analysis hysterectomy show no relationship with epithelial ovarian cancer (OR = 0.97, 95% CI = 0.81–1.14), which was not consistent with the results of Jordan. The reasons of the controversial results include study design of eligible studies, the number of objects in each study, adjustment of confounding factors and the quality of study. In the study of Jordan et al., five cohort studies, 13 case-control studies, one nested case-control study and one pooled analysis of case-control studies were included, whereas, 22 case-control publications of high quality were eligible in this meta-analysis. Furthermore, the previous pooled analysis reported cases diagnosed during 1971–2005, whereas our study was during 1988–2016, we use the newest data available that made the results more persuasive. The result that hysterectomy was associated with a risk of EOC in some studies is reasonable when considered that the most commonly hormone replacement therapy (HRT) of women had a hysterectomy is prescribed oestrogen without a progestin and oestrogen-only HRT could increase the risk of EOC [14].

Cibula et al. [10] found that tubal ligation reduced the risk of EOC by 34% (OR = 0.66, 95% CI = 0.60–0.73). The outcomes in this meta-analysis suggested that tubal ligation was a protective factor of EOC (OR = 0.70, 95% CI = 0.60–0.78), which was consistent with the results of studies above. In the studies of Cibula et al., the data were extract from cohort studies and case-control studies, standard of subgroup analyses and confounding factor were different from our study, most objects were from Asian or the USA. Although their data sources are different from our study, we get the same conclusion, which further confirmed that tubal ligation is a quite possibly protective factor of OC. Tubal ligation may form a mechanical barrier to cut down the retrograde transport of cancerogenic substances via the perineum and vagina [14,54]. The carcinogenic mechanism above provide theoretical support for our research results.

In our study, we used Stata. 12.0 to analyze the data extracted from 38 articles. The results showed that there was high heterogeneity among the included studies. To explore the source of the heterogeneity, we wanted to conduct subgroup analysis based on histological type, as studies showed that showed that the association between endometriosis and EOC risk varied significantly by histologic type [10,41], however, data was limited, we could not extract enough data to conduct an effective meta-analysis. Then, we conducted a subgroup analysis based on area, for the reason that the 38 included publications are mainly conducted in North America, Europe, Oceania and Asia and we wanted to compare the differences among the four areas. The results showed that the subgroup analysis about the association between the three factors and EOC based on area were all the same with the overall results except the Asia group for the association between tubal ligation and EOC (OR = 0.85, 95% CI = 0.58, 1.24), indicating that there was no association between tubal ligation and EOC for Asia.

However, this meta-analysis still has several limitations. Firstly, only those papers written in English were included, relevant articles using other languages had not been enrolled in, leading information loss. Secondly, all studies we searched were published articles, as a result, unpublished data were not available, some useful information may be lost with much possible, what’s more, Deeks’ funnel plot asymmetry test showed that no significant publication bias among the studies, but the bias caused by those unpublished data should not be completely ignored. Thirdly, despite of the strict inclusive criteria, significant heterogeneity was still detected among different studies, we are clear that the association between endometriosis, hysterectomy, tubal ligation and EOC varied significantly, but we couldn’t conduct a subgroup analysis because histological data were limited. Only several studies among the included articles did histology analysis, so we could not extract enough data to conduct an effective meta-analysis.

## 5. Conclusions

The results showed that endometriosis was a risk factor of epithelial ovarian cancer whereas tubal ligation was associated with a reduced risk of epithelial ovarian cancer, hysterectomy may had no relationship with epithelial ovarian cancer.

## Figures and Tables

**Table 1:** Characteristics of 38 case-control publications in this meta-analyses.

Reference	Authors	Country	Group	Year	Case	Control	Risk Factors	NOS Score
[5]	Ness et al.	USA	North America	1999	767	1367	Endometriosis	8
[6]	Madsen et al.	Denmark	Europe	2014	13,241	194,689	Endometriosis	8
[7]	Collette et al.	USA	North America	2014	194	388	Endometriosis	7
[11]	Baandrup et al.	Denmark	Europe	2013	3471	50,576	Endometriosis	8
[14]	Ruiz et al.	USA	North America	2016	208	224	Endometriosis	8
[18]	Modugno et al.	USA	North America	2004	2098	2953	Endometriosis	8
[19]	Nagle et al.	Australia	Oceania	2008	232	1508	Endometriosis	8
[20]	Rossing et al.	USA	North America	2008	812	1313	Endometriosis	8
[21]	Wu et al.	USA	North America	2009	609	688	Endometriosis	7
[22]	Moorman et al.	USA	North America	2009	857	1057	Endometriosis	6
[23]	Bodmer et al.	Switzerland	Europe	2011	1611	9710	Endometriosis	8
[24]	Merritt et al.	USA	North America	2013	1571	2100	Endometriosis	7
[11]	Baandrup et al.	Denmark	Europe	2013	3471	50,576	Hysterectomy	8
[12]	Le et al.	Canada	North America	2014	607	334	Hysterectomy	8
[14]	Ruiz et al.	USA	North America	2016	208	224	Hysterectomy	8
[18]	Modugno et al.	USA	North America	2004	2098	2953	Hysterectomy	8
[19]	Nagle et al.	Australia	Oceania	2008	232	1508	Hysterectomy	8
[22]	Moorman et al.	USA	North America	2009	857	1057	Hysterectomy	6
[24]	Merritt et al.	USA	North America	2013	1571	2100	Hysterectomy	7
[25]	Whittemore et al.	USA	North America	1988	188	539	Hysterectomy	7
[26]	Hartge et al.	USA	North America	1988	296	343	Hysterectomy	6
[27]	Booth et al.	England	Europe	1989	235	451	Hysterectomy	7
[28]	Irwin et al.	USA	North America	1991	494	4238	Hysterectomy	8
[29]	Parazzini et al.	Swizerland	Europe	1993	953	2758	Hysterectomy	7
[30]	Cramer et al.	USA	North America	1995	450	454	Hysterectomy	7
[31]	Rosenblatt et al.	USA	North America	1996	393	2563	Hysterectomy	8
[32]	Green et al.	Australia	Oceania	1997	824	855	Hysterectomy	7
[33]	Wittenberg et al.	The Netherlands	Europe	1999	322	426	Hysterectomy	7
[34]	Riman et al.	Sweden	Europe	2002	655	3899	Hysterectomy	8
[35]	Chiaffarino et al.	Italy	Europe	2005	1031	2411	Hysterectomy	8
[36]	Mills et al.	USA	North America	2005	256	1122	Hysterectomy	7
[37]	Risch et al.	USA	North America	2006	490	534	Hysterectomy	7
[38]	Faber et al.	Denmark	Europe	2013	554	1564	Hysterectomy	7
[39]	Rice et al.	USA	North America	2013	2265	2333	Hysterectomy	8
[6]	Madsen et al.	Denmark	Europe	2014	13,241	194,689	Tubal ligation	8
[7]	Collette et al.	USA	North America	2014	194	388	Tubal ligation	7
[9]	Pike et al.	USA	North America	2004	477	660	Tubal ligation	7
[11]	Baandrup et al.	Denmark	Europe	2013	3471	50,576	Tubal ligation	8
[13]	Huang et al.	China	China	2015	174	70,085	Tubal ligation	7
[14]	Ruiz et al.	USA	North America	2016	208	224	Tubal ligation	8
[18]	Modugno et al.	USA	North America	2004	2098	2953	Tubal ligation	8
[19]	Nagle et al.	Australia	Oceania	2008	232	1508	Tubal ligation	8
[21]	Wu et al.	USA	North America	2009	609	688	Tubal ligation	7
[24]	Merritt et al.	USA	North America	2013	1571	2100	Tubal ligation	7
[30]	Cramer et al.	USA	North America	1995	450	454	Tubal ligation	7
[31]	Rosenblatt et al.	USA	North America	1996	393	2563	Tubal ligation	8
[33]	Wittenberg et al.	The Netherlands	Europe	1999	322	426	Tubal ligation	7
[34]	Riman et al.	Sweden	Europe	2002	655	3899	Tubal ligation	8
[37]	Risch et al.	USA	North America	2006	490	534	Tubal ligation	7
[38]	Faber et al.	Denmark	Europe	2013	554	1564	Tubal ligation	7
[39]	Rice et al.	USA	North America	2013	2265	2333	Tubal ligation	8
[40]	Mori et al.	Japan	Asia	1988	56	112	Tubal ligation	8
[41]	Purdie et al.	Australia	Oceania	1995	824	860	Tubal ligation	7
[42]	Narod et al.	Canada	North America	2001	232	232	Tubal ligation	8
[43]	Mills et al.	USA	North America	2004	256	1122	Tubal ligation	7
[44]	McGuire et al.	USA	North America	2004	417	568	Tubal ligation	7
[45]	Moorman et al.	USA	North America	2008	896	967	Tubal ligation	7
[46]	Grant et al.	USA	North America	2010	495	1086	Tubal ligation	8
[47]	Vitonis et al.	USA	North America	2011	1098	1363	Tubal ligation	8

**Table 2:** Stratified meta-analyses of the association between three risk factors and ovarian cancer.

Risk Factors	N	OR	95% CI	I^2^ (%)	*p*	E-T^2^
**Endometriosis**						
All	12	1.42	1.28–1.57	20.40	˂0.01	0.24
Area						
North America	7	1.42	1.27–1.60	0.00	˂0.01	
Europe	4	1.28	1.12–1.47	0.00	˂0.01	
Oceania	1	2.52	1.63–3.90	--	˂0.01	
**Hysterectomy**						
All	22	0.97	0.81–1.14	84.70	0.68	0.31
Area						
North America	13	1.00	0.93–1.09	74.60	0.94	
Europe	7	0.97	0.86–1.10	88.30	0.63	
Oceania	2	0.98	0.79–1.21	97.40	0.84	
**Tubal ligation**						
All	25	0.68	0.59–0.79	90.50	˂0.01	0.64
Area						
Asia	2	0.85	0.58–1.24	71.80	0.06	
North America	16	0.68	0.64–0.72	87.90	˂0.01	
Europe	5	0.82	0.75–0.89	77.30	˂0.01	
Oceania	2	0.48	0.39–0.60	0.00	˂0.81	

Heterogeneity assumption was checked by I^2^; N, number of studies; E-T^2^, *p*-value of Egger’s test.

## Supplementary Materials

The following are available online at www.mdpi.com/1660-4601/13/11/1138/s1. Figure S1: (A) The subgroup-analysis forest plot for the association between endometriosis and EOC; (B) The subgroup-analysis forest plot for the association between hysterectomy and EOC; (C) The subgroup-analysis forest plot for the association between tubal ligation and EOC, Figure S2: (A) The funnel plot for the association between endometriosis and EOC; (B) The funnel plot for the association between hysterectomy and EOC; (C) The funnel plot for the association between tubal ligation and EOC, Figure S3. (A) The sensitivity analysis plot for the association between endometriosis and EOC; (B) The sensitivity analysis plot for the association between hysterectomy and EOC; (C) The sensitivity analysis plot for the association between tubal ligation and EOC, Figure S4. (A) The cumulative analysis plot for the association between endometriosis and EOC; (B) The cumulative analysis plot for the association between hysterectomy and EOC; (C) The cumulative analysis plot for the association between tubal ligation and EOC.
