# Supplementary material for: The Association between Endometriosis, Tubal Ligation, Hysterectomy and Epithelial Ovarian Cancer: Meta-Analyses
Source: Int J Environ Res Public Health. 2016 Nov 14;13(11):1138. doi: 10.3390/ijerph13111138 (PMC5129348; doi:10.3390/ijerph13111138)
Supplement: Supplementary file 1 [file ijerph-13-01138-s001.pdf]

Supplementary Materials: The Association between Endometriosis, Tubal Ligation, Hysterectomy and Epithelial Ovarian Cancer: Meta-Analyses

Chunpeng Wang, Zhenzhen Liang, Xin Liu, Qian Zhang and Shuang Li

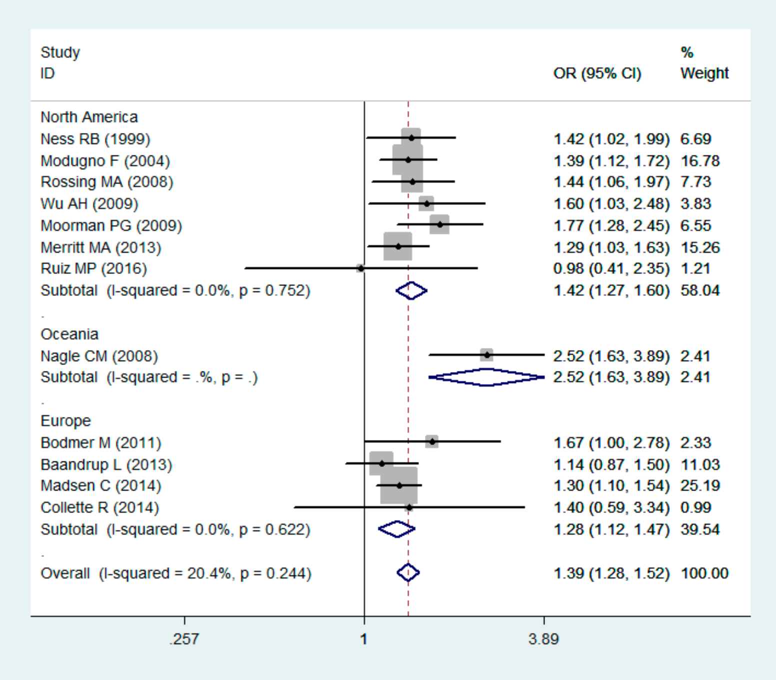

(**A**)

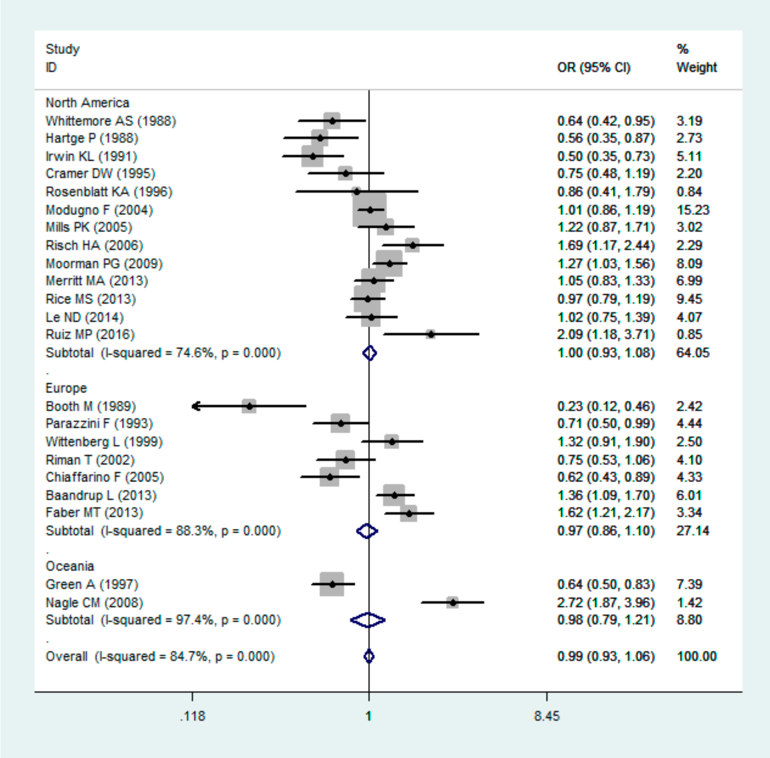

(**B**)

Figure S1. *Cont.*

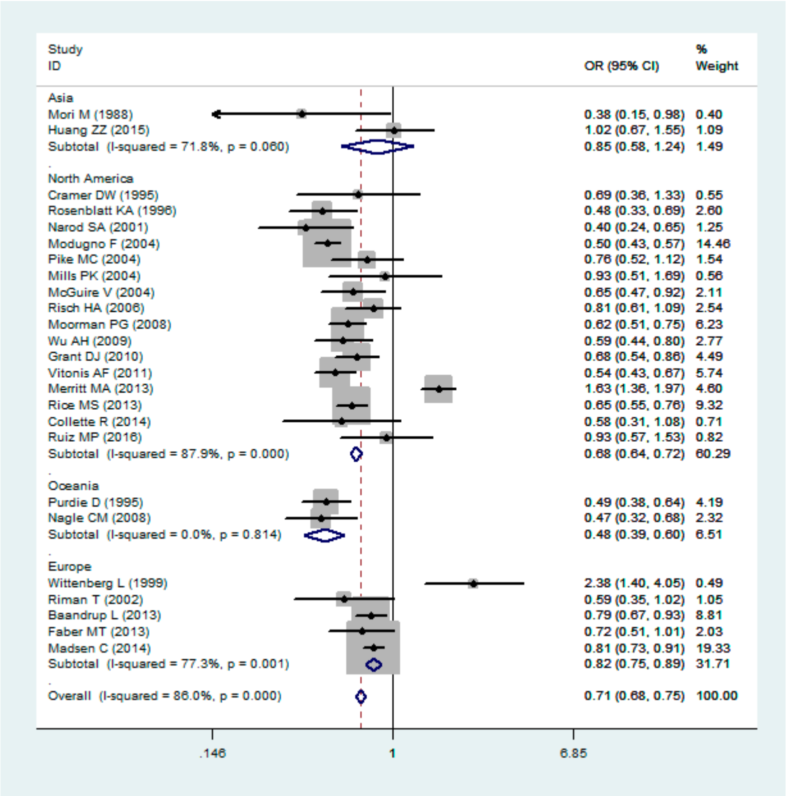

(**C**)

Figure S1. (**A**) The subgroup-analysis forest plot for the association between endometriosis and EOC; (**B**) The subgroup-analysis forest plot for the association between hysterectomy and EOC; (**C**) The subgroup-analysis forest plot for the association between tubal ligation and EOC.

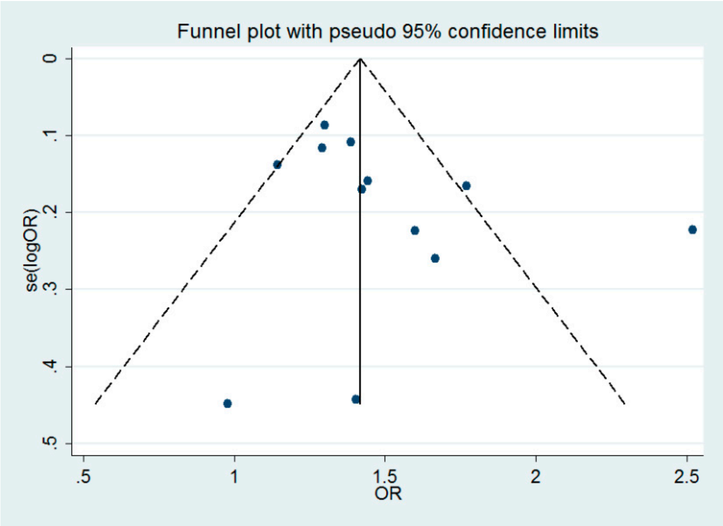

(**A**)

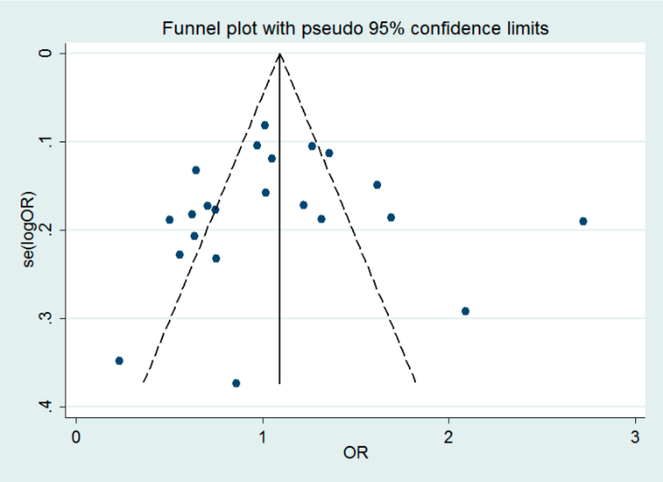

(**B**)

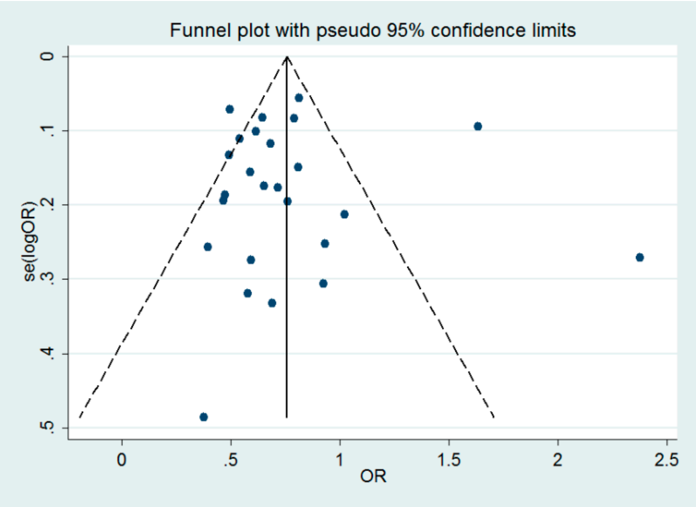

(**C**)

Figure S2. (**A**) The funnel plot for the association between endometriosis and EOC; (**B**) The funnel plot for the association between hysterectomy and EOC; (**C**) The funnel plot for the association between tubal ligation and EOC.

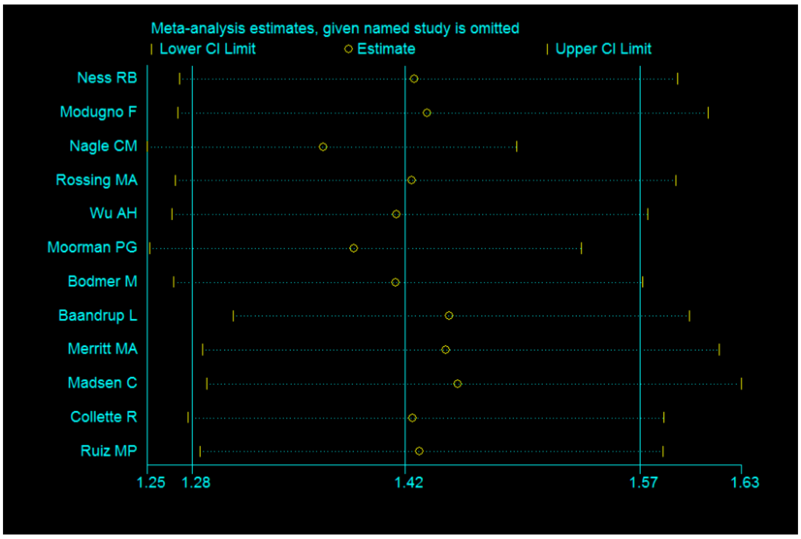

(**A**)

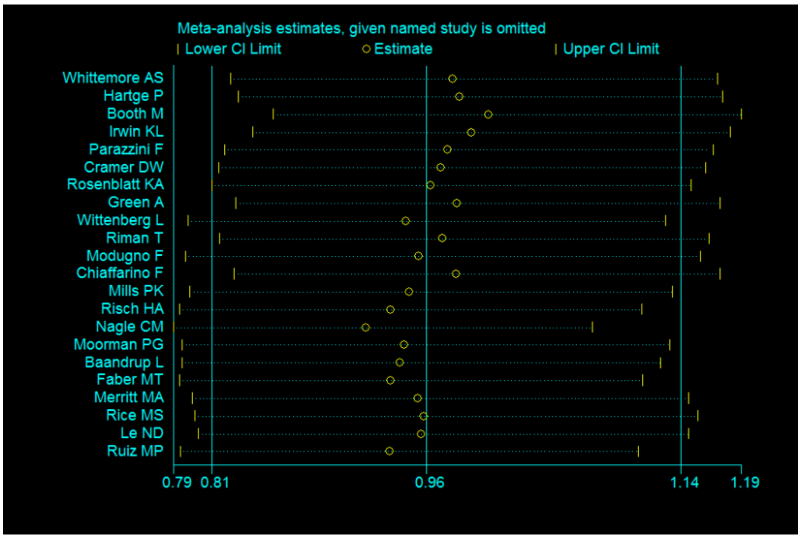

(**B**)

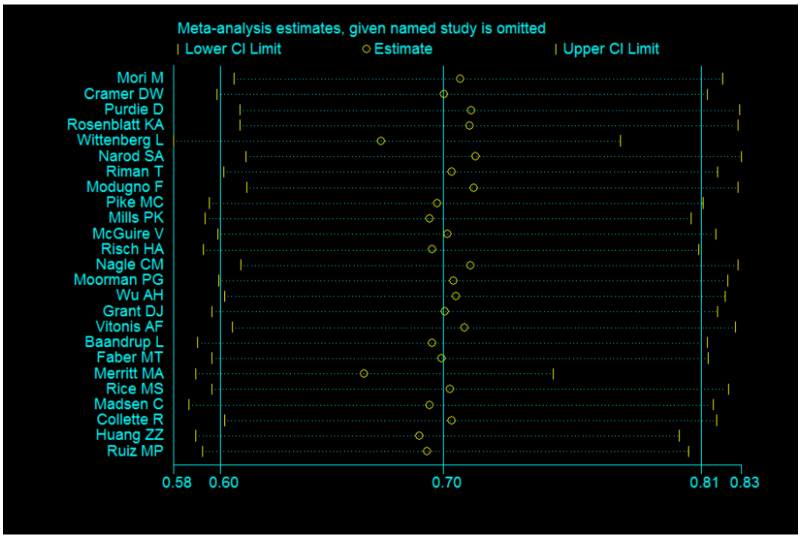

(**C**)

Figure S3. (**A**) The sensitivity analysis plot for the association between endometriosis and EOC; (**B**) The sensitivity analysis plot for the association between hysterectomy and EOC; (**C**) The sensitivity analysis plot for the association between tubal ligation and EOC.

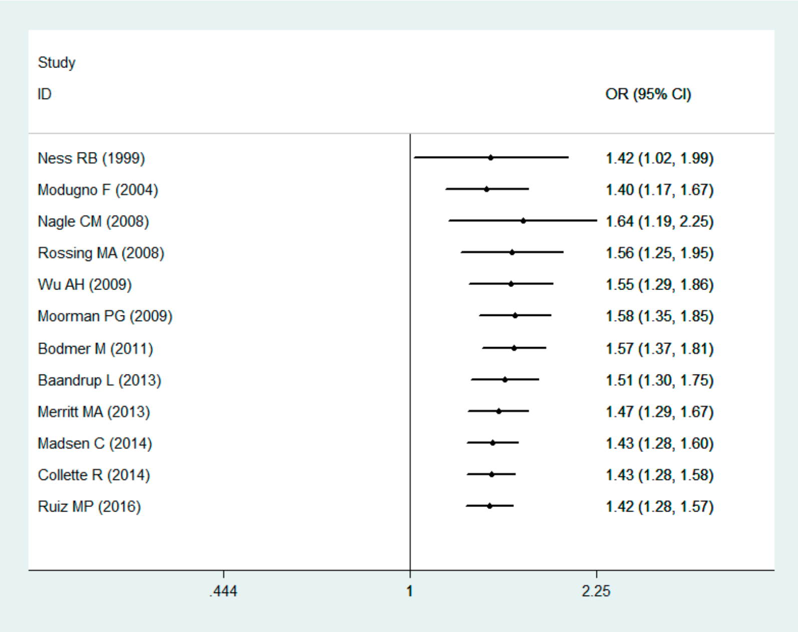

(**A**)

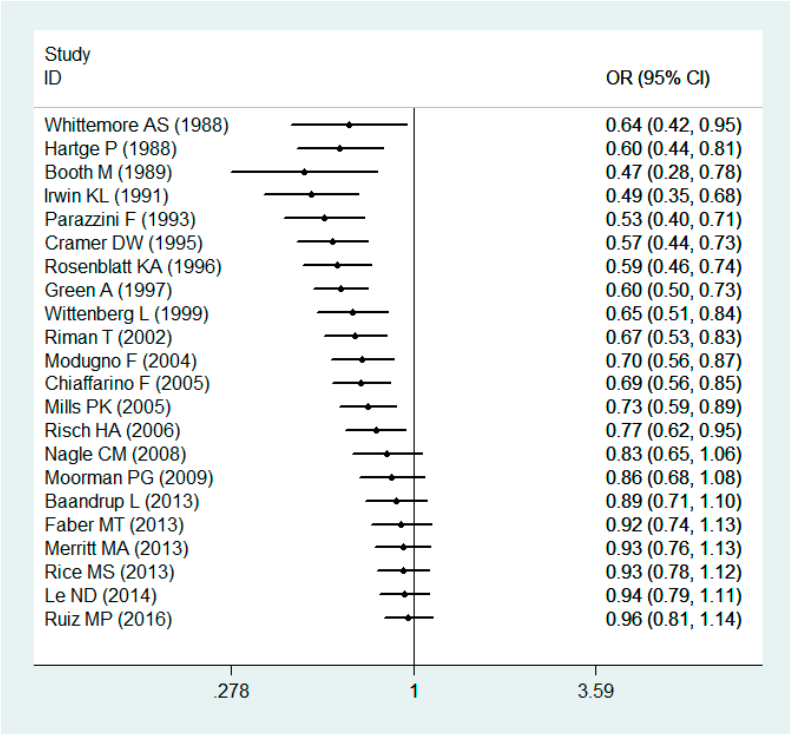

(**B**)

Figure S4. *Cont.*

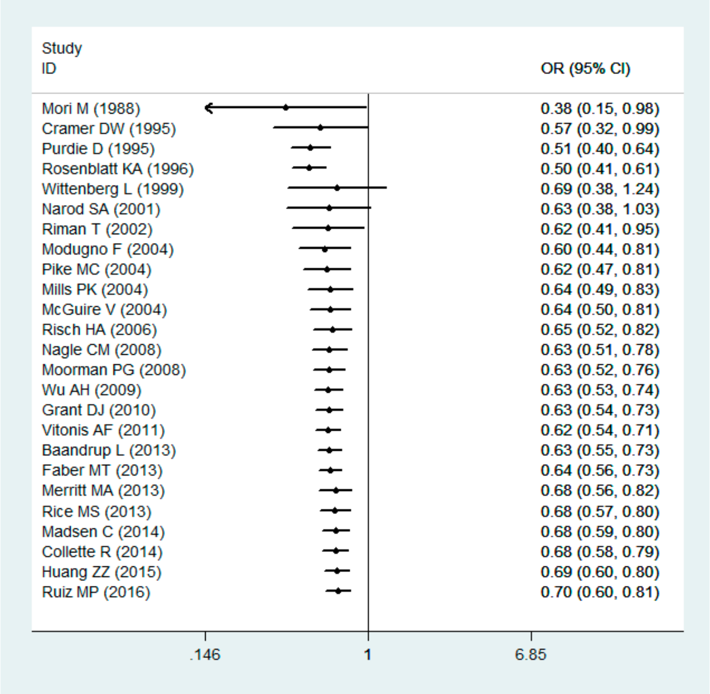

(**C**)

Figure S4. (**A**) The cumulative analysis plot for the association between endometriosis and EOC; (**B**) The cumulative analysis plot for the association between hysterectomy and EOC; (**C**) The cumulative analysis plot for the association between tubal ligation and EOC.

© 2016 by the authors; licensee MDPI, Basel, Switzerland. This article is an open access article distributed under the terms and conditions of the Creative Commons by Attribution (CC-BY) license (http://creativecommons.org/licenses/by/4.0/).
